# What are the barriers to scaling up health interventions in low and middle income countries? A qualitative study of academic leaders in implementation science

**DOI:** 10.1186/1744-8603-8-11

**Published:** 2012-05-29

**Authors:** Gavin Yamey

**Affiliations:** 1 Evidence to Policy initiative (E2Pi), Global Health Group, University of California San Francisco, 50 Beale St, Suite 1200, Box 1224, San Francisco, CA, 94105, USA

## Abstract

**Background:**

Most low and middle income countries (LMICs) are currently not on track to reach the health-related Millennium Development Goals (MDGs). One way to accelerate progress would be through the large-scale implementation of evidence-based health tools and interventions. This study aimed to: (a) explore the barriers that have impeded such scale-up in LMICs, and (b) lay out an “implementation research agenda”—a series of key research questions that need to be addressed in order to help overcome such barriers.

**Methods:**

Interviews were conducted with fourteen key informants, all of whom are academic leaders in the field of implementation science, who were purposively selected for their expertise in scaling up in LMICs. Interviews were transcribed by hand and manually coded to look for emerging themes related to the two study aims. Barriers to scaling up, and unanswered research questions, were organized into six categories, representing different components of the scaling up process: attributes of the intervention; attributes of the implementers; scale-up approach; attributes of the adopting community; socio-political, fiscal, and cultural context; and research context.

**Results:**

Factors impeding the success of scale-up that emerged from the key informant interviews, and which are areas for future investigation, include: complexity of the intervention and lack of technical consensus; limited human resource, leadership, management, and health systems capacity; poor application of proven diffusion techniques; lack of engagement of local implementers and of the adopting community; and inadequate integration of research into scale-up efforts.

**Conclusions:**

Key steps in expanding the evidence base on implementation in LMICs include studying how to: simplify interventions; train “scale-up leaders” and health workers dedicated to scale-up; reach and engage communities; match the best delivery strategy to the specific health problem and context; and raise the low profile of implementation science.

## Background

Most developing countries are currently not on track to reach the health-related Millennium Development Goals (MDGs) [1,2]. A major reason for their slow progress is the “know-do gap”—the gap between what is known and what gets implemented in low and middle income countries (LMICs) [3].The burden of illness in developing countries could be reduced substantially if this gap was narrowed, i.e., if evidence-based tools and services were scaled up. Up to 70% of deaths of children under 5 years, for example, could be prevented through the large-scale implementation of evidence-based interventions [4,5]. In discussing how to improve the health of the world’s poor, McCannon and colleagues argue that “many sound (even powerful) solutions exist, such as new medicines and innovations in health care delivery, but their adoption is unreliable and slow” [6].

The *interventions* that would help LMICs reach the health MDGs, referred to in this paper as “global health interventions,” are well defined. For example, The Bellagio Child Survival Study Group identified 23 evidence-based preventive and therapeutic interventions that could reduce child mortality in LMICs [4]. However, while there is a wealth of evidence on the efficacy and effectiveness of such tools, there has been much less attention paid to how to *deliver* them to scale [7-10]. Whitworth and colleagues frame the issue bluntly: “we do not know how best to scale up interventions effectively” [10].

Insecticide-treated bed nets are a case in point. Such nets reduce childhood malaria episodes by 50% and malaria deaths by 20% [11]. And yet increasing the proportion of children in malaria-endemic countries who consistently sleep under a net has remained a difficult global health challenge, and there is still “considerable debate about how best to deliver nets” [12]. Delivering evidence-based health interventions to the very poorest and most remote communities in LMICs has proven to be a particularly stubborn problem [13,14].

This study aimed to identify some of the key barriers to scaling up health interventions in LMICs through interviews with experts in large-scale implementation, many of whom have led successful national or global health scale-up campaigns, and all of whom have research expertise in implementation science. The study also aimed to identify some of the key unanswered research questions that would help to advance the field of implementation science in LMICs. The term “scaling up” is now widely used in global public health discussions, although there is still no agreed definition. In this paper, I use a working definition proposed by Mangham and Hanson, in which scaling up is described as “the ambition or process of expanding the coverage of health interventions” [15].

## Methods

I contacted 14 public health professionals, purposively selected for their expertise in implementing evidence-based interventions at scale. These professionals were chosen based on a combination of (a) their current or previous experience in leadership roles at institutions that scale up health interventions in LMICs (even if the institutions themselves are or were based in high-income countries [HICs]), and (b) their academic leadership in the field of implementation science. The key informants (KIs) were found through my own personal network of contacts in global health research and practice (I currently lead a university-based global health policy initiative), supplemented by the technique of snowballing [16].

All 14 KIs agreed to be interviewed (they are identified as KIs 1–14 in the Results section of the paper). Thirteen have expertise in scaling-up health tools or services in LMICs, and one focuses on such scale-up in HICs. At the time that the interviews were conducted, three were based in LMICs, 9 in HICs, and two divided their time between LMICs and HICs. Table 1 gives their basic demographic information, outlining their current position; their previous experience relevant to scale-up; the type of intervention that they have been involved in scaling up or have studied (e.g., simple or complex, clinical or public health, and individual or bundled interventions); and their research background. In order to protect KIs’ anonymity, no identifying information is included in Table 1 or in the Results section of this paper.

**Table 1:** Basic demographic information about key informants (to protect KIs’ anonymity, identifying information has been removed)

**Key Informant (KI)**	**Current position**	**Previous experience relevant to scale-up**	**Type of intervention scaled up or studied (in current or past positions)**	**Papers indexed in PubMed: No. of papers, and key topics of research**
KI 1	Academic global health post in a HIC; university leadership post	Leadership of a multilateral health agency, headquartered in Europe; has led large-scale implementation in LMICs	Experience in scaling up both simple interventions (e.g. a specific drug treatment) and complex interventions (e.g. complex public health promotion)	Approx. 300 papers; communicable and non-communicable disease control
KI 2	Academic global health post in a HIC; university leadership post	Previous academic posts; leadership positions in two multilateral organizations, one headquartered in Europe and the other in the US; has led large-scale implementation in LMICs	Experience in scaling up both simple and complex interventions	Approx. 100 papers; global health financing, child health, public health, and communicable and non-communicable disease control
KI 3	Academic global health post in a HIC; leads health service scale-up projects in LMICs	Has led multiple health systems improvement projects in LMICs	Experience in scaling up complex, health systems interventions in LMICs	Approx. 75 papers; scaling up health systems improvements in LMICs, financing of global health
KI 4	Academic public health post in a MIC in Africa; advises national government on large-scale implementation	Research on scaling up in LMICs, with a focus on building research capacity and on health systems	Research on scaling up simple and complex interventions	Approx. 30 papers; implementation science in LMICs, global health research prioritization
KI 5	Academic health policy post in a HIC; specializes in health systems reform	Research on health systems reform in both LMICs and HICs	Research on scaling up complex health systems improvements	Approx. 75 papers; health systems financing in HICs and LMICs, measurement of health system performance
KI 6	Academic global health post in a HIC; university leadership post	Senior positions in national public health service in a HIC	Research on, and experience in, scaling up complex interventions to control risk factors for non-communicable diseases	Approx. 90 papers; risk factors for non-communicable diseases, environmental health interventions, global health diplomacy
KI 7	Academic post in global health in a HIC; university leadership post	Academic leadership positions at national agencies in a HIC that focus on global health problems	Research on scaling up infectious disease control interventions in LMICs	Approx. 200 papers; infectious disease control, global health initiatives, health systems strengthening in LMICs
KI 8	Academic public health post; principal of a medical school in a LIC in Africa	Led national scale-up campaigns addressing communicable diseases	Research on, and experience in, scaling up simple and complex interventions to control communicable diseases	Approx. 150 papers; control of communicable diseases in LMICs, global health training, health systems strengthening
KI 9	Academic public health post at a school of global health in a HIC	Research on large-scale implementation, with a focus on communicable diseases; previous senior positions in regional public health service	Research on scaling up simple and complex interventions to control communicable diseases	Approx. 120 papers; infectious disease epidemiology and control, public health research methods
KI 10	Women’s health researcher in a MIC in Latin America	Academic research posts in LIC universities; leadership of a regional research and implementation initiative	Research on, and experience in, scaling up individual and bundled clinical interventions	Approx. 120 papers; scaling up women’s health interventions in LMICs
KI 11	Implementation science researcher in a HIC	Academic public health posts, with a focus on implementation of health interventions in a HIC	Research on, and experience in, scaling up simple and complex clinical and public health interventions	Approx. 50 papers; implementation science, quality improvement in health care
KI 12	Director of a scale-up program in a LIC	Background in clinical medicine and global public health, dividing time between a LIC and a HIC	Experience in scaling up simple and complex interventions in an LIC	3 papers; scaling up health system interventions in LICs
KI 13	Professor of epidemiology, with a focus on global public health, based at a university in a HIC	Clinical trials, and public health research related to women’s and children’s health	Research on scaling up simple and complex clinical and public health interventions	Approx. 150 papers; scaling up women’s and children’s health interventions, global public health
KI 14	Senior leadership position at an NGO that manages large-scale implementation in LMICs	Past background in business and technology; senior role in a major global initiative to rapidly scale up a communicable disease intervention	Experience in scaling up simple and complex interventions in HICs and LMICs	4 papers; large scale health improvement

I conducted three interviews in person and eleven by phone. Interviews lasted 30–60 minutes, based on a semi-structured questionnaire. Questions were aimed at eliciting interviewees’ personal knowledge, “real world” experience, and knowledge of the implementation science literature. For example, one of the questions was: *“Based on your own personal experience and expertise, what are your own beliefs about why scale up fails?”* Additional file 1 shows the semi-structured interview guide. Interviews were transcribed by hand and were manually coded to look for emerging themes related to my two research questions: What are the some of the key barriers to scaling up health interventions in LMICs? What are some of the key unanswered research questions that would help to improve the science of scale-up in LMICs? Coding was conducted using a constant comparative method [17].

**Figure 1:**
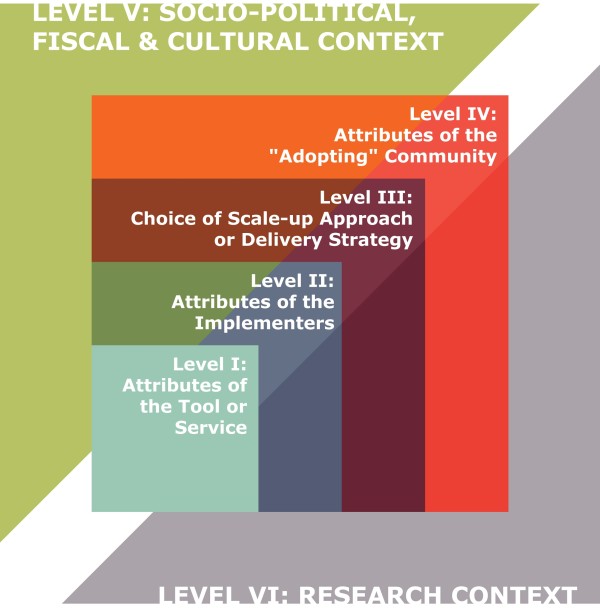
Framework for categorizing the study results.

I organized the results (i.e., the emerging themes on barriers and unanswered research questions) into six categories, representing different components of the scaling up process: (1) attributes of the tool or service being scaled up; (2) attributes of the implementers; (3) choice of scale-up approach or delivery strategy; (4) attributes of the “adopting” community; (5) the socio-political, fiscal, and cultural context; and (6) the research context (Figure 1). These categories were adapted from two previous typologies of scaling up—Hanson and colleagues’ typology of constraints to scaling up [18], and Simmons and Shiffman’s typology of components affecting scale-up success [19].

## Ethics approval

This study was approved by the research ethics committee of the London School of Hygiene & Tropical Medicine. KIs gave their written informed consent for anonymized quotations to be included in this paper. Any information that would identify the KIs was removed from the paper prior to publication to protect KIs’ anonymity.

## Results

### Attributes of the tool or service being scaled up

#### Complexity and lack of consensus

An important theme emerging from the key informant interviews was that overly complex interventions are difficult to scale up in LMICs (KIs 1–3, 14), particularly if there is a lack of consensus about how to scale up that intervention.

*“For many [interventions], that simplicity of consensus isn’t there. For example, water purification or clean water interventions—How to scale up? What kind? What quality? What quantity? Should we involve the private sector? We don’t see progress because there’s no consensus on these questions.”* (KI 2)

Key informants argued that an important avenue for future research is on simplifying interventions for scale-up. One KI mentioned the DART (Development of AntiRetroviral Therapy) trial, which showed that anti-retroviral therapy (ART) can be delivered safely *without* costly, complicated laboratory monitoring, as an example of the type of research that can help improve scale-up:

*“For example, the DART trial asked, ‘how do we simplify ART regimes?’ The KISS principles—keep it short and simple—are very important*.*”* (KI 1)

### Attributes of the implementers

#### Limited capacity

Insufficient capacity in LMICs—human resource, leadership and management, and health systems capacity—emerged in the interviews as a key barrier to implementing evidence-based interventions at scale (KIs 2, 4–9). KIs mentioned research on expanding the workforce and on engaging private actors, such as non-governmental organizations [NGOs] and drug store owners, in scale-up processes as important topics for implementation research (KIs 6,12).

*“Human resource deficiencies are a barrier—not just numbers, but a sense of being able to do things. There’s a brain drain. Good people leave, siphoned off by NGOs. Human resource deficiency is a critical issue.”* (KI 6)

*“On the major workforce questions—how do we have a bigger one? Keep people in place? Use people better?—few solutions are evidence-based, except for task shifting.”* (KI 12)

A related barrier identified in this study was weak leadership, governance, or management capacity, especially at the local level (KIs 2, 5, 7). Key informants suggested that an important research question for improving scale-up is whether it is possible to identify and foster public health leaders (KIs 5,8,12,13).

*“Leadership is hugely important. Leadership is a major issue. Lots of systems have tried reengineering with poor results, often because local and national leadership are missing.”* (KI 5)

*“Is leadership transferable or must you live with it or without it? Leadership really does matter—but can youmakebetter leaders?”* (KI 5)

KIs also identified weak *health systems* as one of the main bottlenecks to scaling up proven interventions and to reaching the MDGs (KIs 5, 12).

*“There are operational constraints at the local level—manpower, buildings, the local constraints can vary. You need local adoption, but there are local obstacles.”* (KI 5)

A final research question that emerged from the interviews, related specifically to the implementers themselves, was the question of how best to incentivize provider behavior towards implementing interventions to scale (KIs 3,5,13).

*“How do you build in systems and incentives to successfully build in implementation and sustain it down the road? That question is never going to leave. There’s interesting research around how you keep it going.”* (KI 3)

### Choice of scale-up approach or delivery strategy

#### Failure to actively spread knowledge of an innovation

Key informants suggested that scale-up of a health innovation may fail when implementers do too little to actively spread that knowledge (KI 3,10).

*“We know that knowledge [alone] does not produce change. We needmorethan knowledge.”* (KI 10)

### ***Poor application of diffusion techniques***

The science of large scale implementation draws upon Rogers’ work on diffusion of innovations (KI 5), including the use of targets and incentives (KIs 5, 11). But KIs argued that these techniques need to be applied at the right time to the right “unit” of the health system, or else diffusion is unlikely to occur (KIs 5, 11, 14).

*“One of the major barriers is that the wrong approach to dissemination is used at the wrong moment in time”* (KI 14)

*“One area I’ve worked in is public health targets in low income countries and high income countries. A very frequent complaint is that these get nowhere. They’re statements of wish rather than instruments for achieving change. There’s a lack of sustained attention at the national level, a lack of incentives for people at the sharp end of change, a lack of information, a lack of comparators to know whether you’re lagging behind.”* (KI 5)

### ***Non-transferability and non-scalability from trial conditions***

Several KIs argued that a key reason why an intervention that was successful in a pilot may not scale up in a “real world” setting is diminished implementation fidelity—that is, in the real world the intervention is not delivered as intended (KIs 4, 5, 12, 14). The KIs argued that such fidelity can be diminished by misjudging the readiness of an intervention to go to scale and by differences between the conditions under which the pilot was conducted and the scale-up context (KIs 4, 5, 12, 14).

*“RCTs [randomized controlled trials] are done under specific conditions; you’re then transferring it [the intervention] into a different context, with a different case mix, different demographics, different transportation.”* (KI 4)

*“It’s assumed that success in one village will work elsewhere—it’s a naiveté to exponential scaling. Some things will scale, some things won’t*.” (KI 12)

*“People go to scale prematurely; the focus has been on efficacy, not effectiveness.”* (KI 14)

A key area of future research identified by the KIs is research that would help implementers to better understand the local context (KIs 1,7,8,13):

*“Scale-up is context-specific. We need to understand the local context better. The case study method is to implementation science what the RCT is to clinical evidence.”* (KI 1)

### ***Poor models of efficient scale-up approaches***

KIs discussed what they see as a knowledge deficit on successful dissemination strategies (KIs 11–13); one of these KIs said that the result of this deficit is that we do not have good models for “efficient” scale-up:

*“Building good models for scale up? We don’t have the information to do it.”* (KI 12)

### Attributes of the “adopting” community

#### Lack of “community readiness” or engagement

One explanation provided by the KIs for why scale up fails is that the targeted community was not “ready”—there was insufficient community consultation and too little engagement of key community stakeholders (KIs 4,12,14).

*“For example, if you’re changing guidelines for hypertension, who must you consult? Not just policymakers, also clinicians, key opinion leaders on the ground,andthe community itself.”* (KI 4)

KIs discussed the importance of research on how to better engage the community itself in scale up efforts (KIs 7, 10, 13):

*“We need to learn fromusers, not providers. We need to learn from people themselves. We need qualitative research to see howthey can tellus the solutions, so we can work with them.”* (KI 10)

*“What kind of community mobilization works or not?”* (KI 13)

Scaling up, argued two KIs, requires overcoming demand-side barriers (KIs 7, 12). An important avenue of research, they explained, is to understand why communities in LMICs sometimes do not take up services when these are provided and how these “demand side” barriers can be overcome (KIs 7, 12).

*“In scale-up, we often think of thesupplyside, but there’s less emphasis on the demand side. Coke [Coca-Cola] doesn’t scale up unless peopledemandit. To increase births in facilities, women must demand it.”* (KI 12)

### Socio-political, fiscal, and cultural context

#### Financial constraints

Financial constraints at the community, national, and global levels are constraining scale-up of a variety of interventions (KIs 6–8, 12):

*“It is very limiting if there are no funds committed centrally by the government to scale-up.”* (KI 8)

*“There’s a funding gap to scale up services: 10–20 billion dollars that we don’t have*.” (KI 12)

Given these financial constraints, an avenue of research identified by the KIs in this study is on how to reach the poorest and most vulnerable communities with tools and services (KIs 3–4):

*“One of the issues around scaling up is the economic differences between groups where scale-up is happening. Reaching the very poor is not the same as scale-up for higher socioeconomic status. They may all need a vaccine, but scale-up differs for these two groups.”* (KI 4)

The key informants acknowledged that mobilizing new resources for scaling up will be important, but also that research is needed on improving the way in which finances are tracked:

*“This could involve individual citizens or patients—if you have a good, empowered citizenry, they can hold institutions to account, through a consumer process or a democratic process. I’m more persuaded by accountability via the ballet box or markets.”* (KI 5)

### ***Lack of donor coordination***

Although the last decade has seen a rise in international development assistance for health, poor coordination between donors can impede implementation (KIs 6, 12). One KI cited the work of David Fidler on the “anarchy” occurring within the governance of global health assistance, which he said has an impact upon LMICs:

*“The other [barrier to scale-up] is the cacophony and anarchy that David Fidler describes—a lack of donor coordination. It’s very hard for countries to deal with. The World Bank comes in and expects everyone to jump. USAID [the United States Agency for International Development] comes in and expects everyone to jump, and to do so with democratic fervor.”* (KI 6)

### ***Political constraints: Lack of political will and decision-making based on politics rather than science***

KIs said that a lack of political interest in scale up was a major barrier (KIs 12, 14); they mentioned that campaigns to massively scale-up ART in low income countries (such as the WHO’s “3 by 5” campaign, or the US President’s Emergency Plan for AIDS Relief) had helped to generate political interest in large-scale implementation.

*“Until recently, we didn’t have the political interest to scale up.”* (KI 12)

One KI noted that a barrier to implementing evidence-based interventions in LMICs is that decisions get made based on politics, rather than a proper understanding of the data on effectiveness and costs (KI 4). A second KI discussed the politicization of the leadership within ministries of health in LMICs (KI 7).

“Evidence isn’t used. People make decisions based on politics, for example introducing the pneumococcal vaccine with no understanding about its effectiveness or cost-effectiveness.” (KI 4)

*“It [leadership] is frequently politically aligned and not related to health expertise.”* (KI 7)

### ***Regulatory hurdles***

One KI noted that a proven strategy for going to scale with antiretroviral drugs (ARVs) is to allow non-physicians to prescribe them, but in some settings this is forbidden:

*“There are regulatory hurdles for health systems strengthening, such as the way medicines are prescribed or who can prescribe medicines. For example, if the only people who can prescribe ARVs are doctors, that’s a big barrier in the system.”*(KI 4)

### Research context

#### Low profile of implementation science

KIs argued that the low status of implementation science among researchers is a barrier to finding effective ways to deliver interventions to scale, and that raising the status of this science is a key imperative for improving large-scale implementation in LMICs (KIs 2, 7–10, 14).

*“Many researchers don’t see this kind of research as exciting—don’t see it as highly academic or challenging”* (KI 8)

*“There’s no well established field of inquiry—nothing to attract young researchers into the field.”* (KI 14)

### ***Limited research capacity in LMICs***

Two KIs felt that researchers and institutions in LMICs are the ones who are closest to the problems on the ground, yet they are highly constrained in tackling these issues by their limited research capacity (KIs 2, 12):

*“Everybody has the experience of the typical management environment, day to day, in a developing country—it’s hand to mouth, fire fighting, ad hoc decision making. The ability to say ‘let’s pause here and do an operational research study’ seldom happens. There’s no time, no training, no people to do the work.”* (KI 2)

### ***Lack of robust research methods and innovative research designs***

Implementation science is hindered by reliance on uncontrolled retrospective assessments and descriptive studies and a lack of large-scale cluster randomized studies (KIs 4, 12, 13, 14). In addition to using randomization, there is also a need for new study designs (KI 4, 11,12,14), better ways to capture public health data (KI 12), and new ways to synthesize existing evidence (KI 14).

*“The RCT is one way of doing evaluation. It isn’t a bad thing. But maybe there’s an intermediate method needed between an RCT and scale-up.”* (KI 4)

*“Scale-up won’t be successful until we can measure it better.”* (KI 12)

*“Generally there’s a reliance on methods of evaluation or research that don’t help us learn continually as we expand [an intervention]*.*”* (KI 14)

## Discussion

The purpose of this study was to explore, from expert interviews with academic leaders in the field of implementation science, some of the key barriers to the scale-up of proven interventions in LMICs. The study also aimed to define a set of key research questions that need to be addressed in order to help overcome these barriers. The personal views expressed by KIs in this qualitative study are valuable in that they are reflective of their “real world” experience in implementing or researching scale-up processes in the developing world. There are six findings from this study that could have important public health implications.

First, the KIs’ experience suggests that scale-up is less likely if the intervention is highly complex and there is lack of consensus about its value, a finding that is supported by the published implementation science literature [19-22]. For example, a review of scaling up by the World Bank argued that the process of scaling up should be “driven by a universalist process of simplifying rules and procedures for use by many people on a larger scale” [21]. Given that KIs in this study saw complexity as a barrier to scale-up, an important avenue for future research that emerged from the KI interviews was ways to reduce intervention complexity. An example of such research is the landmark DART trial [23], which should facilitate simplified and decentralized delivery of ART in LMICs.

However, there are several global health interventions that are inherently complex to implement, such as the social marketing of condoms and the DOTS (directly observed therapy, short course) strategy for tuberculosis control in very low income countries [24]. It is unlikely that these kinds of complex public health interventions could be dramatically simplified. Gericke et al. have proposed a conceptual framework for characterizing such complexity, which has four dimensions: characteristics of the intervention; characteristics of delivery; requirements on government capacity; and usage characteristics [24]. Mangham and Hanson argue that “understanding intervention complexity,” for example by using Gericke et al’s framework, can help to overcome barriers to scaling up complex interventions, including the important barrier of resource constraints [15].

Second, the study findings suggest that both “leaders and systems” influence scale-up. KIs argued that local and national leaders play a crucial role in ensuring good governance, developing concerted scale-up policies, and getting buy-in for these policies from health workers. Indeed empirical research has shown that strengthening national leadership can increase the likelihood of scale-up success [6,25,26]. For example, in 2002, Egypt’s Ministry of Health and Population, with donor assistance, introduced a Leadership Development Programme in Aswan Governorate, which aimed “to improve health services in three districts by increasing managers' ability to create high performing teams and lead them to achieve results” [26]. The program was associated with an increase in the number of new family planning visits and a fall in the maternal mortality rate compared with control districts. Yet despite the emerging evidence of the importance of strong leadership, there has been little research on identifying and fostering “implementation leaders.”

While leaders play a role in championing a global health intervention, its delivery to scale also needs strong “systems components,” especially a robust healthcare workforce. Many LMICs are currently grappling with a crisis in human resources for health (HRH), caused by factors such as under-investment in health education; the HIV/AIDS epidemic, which has increased the workload on health professionals and exposed them to infection; and brain drain of health professionals [27,28]. The crisis is impeding scale-up of many global health interventions that are critical to reaching the health-related MDGs, including interventions related to HIV, sexual health, maternal health, and mental health [29-39]. The Joint Learning Initiative, a consortium of over 100 global health leaders with expertise in HRH, has stated that "the only route to reaching the health MDGs is through the [health] worker; there are no short cuts" [28].

The specific nature of the HRH crisis varies between countries, for example in the geographic distribution of health workers, the nature of the staff shortages at different levels of the health service, and the proportion of workers who have received high quality training [28,40]. There is therefore no single, simple “blueprint” for solving this crisis—long-term solutions will depend to some extent upon the local context.

The KIs argued that an important research question that could help to address the HRH crisis is how best to train and incentivize a cadre of workers, including community health workers, to deliver the tools needed to reach the health MDGs. Health worker incentives have become an important focus of attention in discussions on the HRH crisis in sub-Saharan Africa [41-43]. Mathauer and Imhoff, for example, argue that “experience and the evidence suggest that any comprehensive strategy to maximize health worker motivation in a developing country context has to involve a mix of financial and non-financial incentives” [41]. Their recent qualitative study of health worker motivation in Kenya and Benin suggested that these non-financial incentives include ensuring that workers have the equipment, supplies, and training that they need to do their job to a high professional standard [41].

Given that the HRH crisis is felt particularly acutely in the public sector, KIs argued that implementation research should also include how best to engage non-state actors, including NGOs and the private sector, in scale-up efforts. Leveraging the non-state health sector, including improving the quality of services provided by private health providers such as through social franchising initiatives, is seen by many commentators as an important mechanism for overcoming barriers to achieving the MDGs [39].

The global campaign to scale up effective malaria drugs—artemisinin-based combination therapies (ACTs)—is a good case in point. Weak public sector health systems, including health worker shortages and weak drug supply chains causing drug stock-outs, have hindered efforts to ensure that all patients with uncomplicated falciparum malaria receive ACTs [44,45]. Makundi and colleagues argue that in Tanzania, “limited human resources for malaria interventions, especially at the district level, impact negatively on the delivery of interventions” [45]. There is growing realization within the global health community that non-state actors, including pharmacies and drug stores, NGOs, and health charities, could play an important role in scaling up ACTs [46,47]. Two recent sub-national trials, one randomized and the other quasi-randomized, showed that introducing an ACT price subsidy into the private sector supply chain can significantly reduce ACT prices in retail stores and increase their use [48,49]. Based on the success of such small pilots, a price subsidy scheme, called the Affordable Medicines Facility-malaria, is currently being piloted at national level in eight LMICs [50].

Third, “engagement matters.” Based on their experiences of successful scale-up, KIs emphasized that global health initiatives are likely to fail unless they engage local implementers and the recipient community itself, an assertion supported by a growing body of research evidence [7,20]. Studies are needed to better understand what makes a community “ready” or “activated” for scale-up, why communities sometimes fail to take up proven interventions, and how such “demand side” barriers can be overcome. Reaching and engaging the poorest communities remains a stubborn problem and an ongoing research imperative [7,13].

Fourth, although there is a large literature on diffusion techniques, including on the diffusion of innovations and the application of social network theories to scale-up, [6,51] nevertheless KIs pointed out that there remain large gaps in our understanding of which techniques work best at which time and which level of the health system. The global health community still lacks good models for efficient scale-up approaches.

Fifth, an important finding emerging from this study is that scaling up an intervention is not an isolated process. Its failure or success is closely tied with a complex array of contextual factors, such as political will, politics, the regulatory environment, the donor environment (including whether donors coordinate their efforts or act in isolation) [52], and the fiscal environment. These can operate at multiple levels of the health system. For example, the research literature suggests that financial constraints to scale-up act at the individual, household, community, national, and international levels [53].

Finally, KIs argued that implementation research plays a valuable role in the process of translating proven interventions into public health gains, and yet such research currently suffers from its low status and priority and its lack of funding. An expert meeting held in 2010 at the Fogarty International Center at the U.S. National Institutes of Health, on “implementation science and global health,” concluded that this type of research could act as “the engine of accelerated progress” [54]. Whitworth and colleagues propose that “strong health research systems and research programmes that address bottlenecks to upscaling effective interventions should be developed without delay” [10].

### Study limitations

To the best of my knowledge, this is the first qualitative study of global health professionals, who are also academic leaders in the field of implementation science, to have explored their views on barriers to large-scale implementation in global health. The KIs’ views on operational successes and failures gave a “pragmatic, real world” dimension to the study results.

Nevertheless, the study has at least three major limitations. First, this was a small qualitative study, with only 14 KIs. Including a larger number of KIs may have led to identification of additional, important themes.

Second, most of the KIs have been affiliated with international health agencies, donor agencies, or research institutions that are headquartered in high-income countries, while only five (KIs 4,8,10,12,14) have been directly involved in delivering or studying implementation *at the local level*. The demographic make-up of the KIs (i.e., their professional background and institutional affiliation) is likely to have influenced their views on the difficulties of scaling up global health interventions. The results of the study may have been different if it had included a broader range of experts in scaling up, including more who are directly involved in local “on the ground” implementation.

Donors and international agencies, for example, who were well represented in this study, tend to have a different approach towards solving global health challenges than local implementers. In malaria control, they tend to favor a “visible quick-fix solution” [55] over more complex approaches. The specific demographic make-up of the KIs may also have played a role in their views on community engagement. Donor agencies have been criticized for viewing community engagement in a “top-down” manner, as a way to increase the chances of donor-led interventions—especially disease-specific, vertical programs—being scaled up [56]. In contrast, local implementers may have a more “bottom up” view, in which local ownership of the intervention is paramount [56]. While donors have publicly agreed to support country ownership of how development assistance is used, as reflected in the Paris Declaration on Aid Effectiveness,[57] a recent study found that donors were doing poorly at living up to the declaration’s principles and are still largely dictating the terms of how such aid is used [58].

Third, the study had only one author. This limitation could have introduced bias into the coding of the transcripts.

## Conclusions

Despite these caveats, the study may be of value to policymakers and researchers tackling implementation problems, because it suggests an initial series of “next steps” in translating knowledge into large-scale change in global health. These include studying ways to: simplify interventions; train future “scale-up leaders”; build and incentivize an implementation workforce; reach and engage recipient communities, especially the poorest; apply the most effective diffusion techniques for scaling up the right tools at the right time in the right place within a health system; and raise the low profile of implementation science. An accompanying article—a short essay—which was also based on these key informant interviews, has laid out some of the “success factors” associated with successful scale-up [59]. The next steps laid out above will be challenging to achieve, but the stakes are high. Without concerted action, argue Sanders and Haines, “the unconscionable gap between knowledge and its implementation will persist in the health field [60].”

## Competing interests

GY leads the Evidence to Policy initiative (http://www.e2pi.org), which has received funding from the Bill & Melinda Gates Foundation, the Global Fund to Fight AIDS, Tuberculosis and Malaria, and the Clinton Health Access Initiative; all three organizations are major funders of global health scale-up programs. From May to August 2012, he was a member of the project team that is conducting an external evaluation of UNITAID, another organization that funds scale-up activities.

## Authors’ contributions

GY designed and conducted the study and wrote the manuscript.

## Supplementary Material

Additional file 1 Semi-structured interview guide. Click here for file
