# Supplementary material for: What are the barriers to scaling up health interventions in low and middle income countries? A qualitative study of academic leaders in implementation science
Source: Global Health. 2012 May 29;8:11. doi: 10.1186/1744-8603-8-11 (PMC3514334; doi:10.1186/1744-8603-8-11)
Supplement: Additional file 1 — Semi-structured interview guide. [file 1744-8603-8-11-S1.doc]

**Additional File 1. Semi-structured interview guide**

For the key informant interviews, I used a semi-structured schedule of questions, as shown below, asking additional questions as prompted by informants’ answers:

1. In your view, what are the key scientific theories that can help explain the reasons why the adoption of effective global health tools (e.g. condoms, bed nets) is often slow and unreliable?
2. Based on your own personal experience and expertise, what are your own beliefs about why such scale up often fails?
3. What do you think are the key scientific theories and models to explain successful scale-up?
4. What are your own beliefs, from your own experience, to explain successful scale-up?
5. What are the key papers that you think helped to inform the science of scale up, including those that are in the grey literature?
6. What are the big gaps in the field of implementation science—what are some of the research questions that need to be addressed to help fill the gaps in the knowledge base on scale up?
